# *In Situ* Spatiotemporal Mapping of Flow Fields around Seeded Stem Cells at the Subcellular Length Scale

**DOI:** 10.1371/journal.pone.0012796

**Published:** 2010-09-17

**Authors:** Min Jae Song, David Dean, Melissa L. Knothe Tate

**Affiliations:** 1 Department of Biomedical Engineering, Case Western Reserve University, Cleveland, Ohio, United States of America; 2 Department of Neurological Surgery, Case Western Reserve University, Cleveland, Ohio, United States of America; 3 Department of Biomedical Engineering, Department of Mechanical and Aerospace Engineering, Case Western Reserve University, Cleveland, Ohio, United States of America; Massachusetts Institute of Technology, United States of America

## Abstract

A major hurdle to understanding and exploiting interactions between the stem cell and its environment is the lack of a tool for precise delivery of mechanical cues concomitant to observing sub-cellular adaptation of structure. These studies demonstrate the use of microscale particle image velocimetry (μ-PIV) for in situ spatiotemporal mapping of flow fields around mesenchymal stem cells, i.e. murine embryonic multipotent cell line C3H10T1/2, at the subcellular length scale, providing a tool for real time observation and analysis of stem cell adaptation to the prevailing mechanical milieu. In the absence of cells, computational fluid dynamics (CFD) predicts flow regimes within 12% of μ-PIV measures, achieving the technical specifications of the chamber and the flow rates necessary to deliver target shear stresses at a particular height from the base of the flow chamber. However, our μ-PIV studies show that the presence of cells *per se* as well as the density at which cells are seeded significantly influences local flow fields. Furthermore, for any given cell or cell seeding density, flow regimes vary significantly along the vertical profile of the cell. Hence, the mechanical milieu of the stem cell exposed to shape changing shear stresses, induced by fluid drag, varies with respect to proximity of surrounding cells as well as with respect to apical height. The current study addresses a previously unmet need to predict and observe both flow regimes as well as mechanoadaptation of cells in flow chambers designed to deliver precisely controlled mechanical signals to live cells. An understanding of interactions and adaptation in response to forces at the interface between the surface of the cell and its immediate local environment may be key for *de novo* engineering of functional tissues from stem cell templates as well as for unraveling the mechanisms underlying multiscale development, growth and adaptation of organisms.

## Introduction

Recent studies demonstrate the promise of delivering spatiotemporally controlled mechanical cues to guide stem cell proliferation patterns [1]–[3] and lineage commitment [1], [2], essentially harnessing nature's approach to engineering tissues. Furthermore, it has recently been shown that embryonic mesenchymal stem cells exhibit 1000-fold greater mechanosensitivity than terminally differentiated cells [4], [5]. However, a major hurdle to understanding and exploiting interactions between the stem cell and its environment is the lack of a tool for precise delivery of mechanical cues concomitant to observation of sub-cellular structural adaptation. On the one hand we can predict flow regimes and observe mechanoadaptation of cells in flow chambers designed for delivery of controlled mechanical signals, using computational fluid dynamics (CFD) and *in situ* microscopy of live cells [1]. Furthermore, microscale particle image velocimetry (μ-PIV) allows for validation of CFD predictions at the length scale of the cover slip onto which cells are seeded for mechanotransduction studies. However it is unknown how well coverslip length scale (diameter 1.5 cm) flow calculations and displacement measures predict cell scale (10–20 µm) flow environments and/or the adaptation of cells in those environments. In the current study we demonstrate and quantify, for the first time to our knowledge, three dimensional flow fields at the length scale of the stem cell in order to determine how well CFD predicts the local mechanical milieu of the cell and to provide a tool for real time observation and analysis of stem cell adaptation to the prevailing mechanical milieu (reported on in a companion study). We hypothesize that CFD provides at least 80% fidelity in predicting the target flow regimes to be delivered to cells, but that the actual fluid drag induced shear stresses experienced at the subcellular scale will be higher than those predicted by CFD due to the effects of cell seeding density as well as distance from the substrate on which cells are seeded.

## Materials and Methods

### Computational Fluid Dynamics

A Computational Fluid Dynamics (CFD) model was built to calculate flow regimes (CFD-ACE, SOLVER, GEOM, and VIEW, ESI group), including velocity, pressure and shear stress distribution on cells within a flow chamber designed to impart highly controlled stresses to cells [1], [6]. Flow was calculated from the continuity equation (1) and Navier-Stokes equation (2) using a 2nd order upwind-discretization scheme in three dimensions. Wall shear stress is calculated from the wall strain rate (3). We assume that the flow medium is incompressible and that flow is laminar at rates of interest for physiological relevance. These assumptions are appropriate, given that the flow medium is similar to 0.9% saline, a Newtonian fluid with density comprising 996 kg/m^3^, at body temperature (310K), and laminar viscosity (0.001kg/ms). The Navier-Stokes equation is applied, assuming that body forces are negligible and that flow is steady in three dimensions, also appropriate assumptions for the length and time scale as well as the flow velocity studied [1], [2]. Hence,
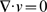
(1)

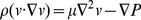
(2)

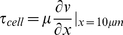
(3)where 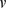 is the velocity vector, 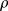 is density, 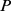 is pressure, 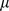 is viscosity, 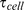 is the shear stress at 10 µm height, 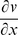 is the strain rate, and 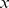 is the height from bottom of chamber.

Flow is induced using an input pressure gradient of 3.9 Pa to achieve a flow rate of 0.13 ml/min, which is necessary to achieve a target shear stress of 0.2 dyn/cm^2^ (0.02 N/m^2^) on the apical surfaces of cells. This shear stress was chosen, because it was shown previously to result in changes in gene expression associated with steering of cell fate during the first stage of skeletogenesis [7], [8]. This CFD model does not account for the presence of cells in the flow chamber, but rather it calculates stresses at a height typical of the cells' apical surface. CFD predictions were then validated using experimental data on an equivalent model system (flow through an actual chamber without cells seeded within). Thereafter experimental studies were carried out to measure actual flow fields in the vicinity of cells.

### Cell preparation and μ-PIV

C3H/10T1/2 cells, a cell line derived from the mesenchyme of the murine embryo (CCL-226; ATCC, Manassas, VA) were passaged until passage 5 or 6 (P5 or P6) in 10 ml of Basal Media Eagle (BME) supplemented with 10% fetal bovine serum, 1% L-Glutamine, and 1% Pen/Strep (Invitrogen, Carlsbad, CA) in T-75 Flasks (Corning, Inc., Corning, NY). Using a hemocytometer, we counted the number of cells in each of four grid regions and averaged the four numbers to determine cell density. From the desired cell density, area of the cell seeding surface, and the suspension volume of each well, we calculated and used the appropriate suspension volume to achieve the target cell density per unit area. Five glass coverslips were treated with radiofrequency glow-discharge (RFGD) to improve cell adhesion to the coverslip in the absence of extracellular matrix proteins which could affect biological and mechanical outcome measures. Then, C3H/10T1/2 cells were seeded on the coverslips at densities including 0, 5,000, 15,000 (a “low density” applied in previous mechanoadaptation studies [7], [8]), 25,000, 35,000, 45,000, and 85,000 (a “very high density” of interest [7], [8] cells/cm^2^). Cells were then incubated for 24 hours prior to flow visualization studies.

Flow regimes were visualized around live cells using micro Particle Image Velocimetry (μ-PIV) methods. Fluorescent microparticles, 1 µm in diameter, were diluted (0.1mL of Tetraspeck (Invitrogen) microparticles suspended in 13 mL of BME supplemented 1% L-Glutamine, and 1% Pen/Strep) to allow for the observation of fluid displacements in time and space, with respect to cells that were fluorescently labeled with calcein green (Invitrogen). Flow was controlled using a syringe pump (Harvard Apparatusn, Holliston, MA) and the flow rate was predicted using CFD to deliver the target 0.2 dyn/cm^2^ shear stress to the apical surface of cells seeded within the chamber. The flow channel dimensions were determined by the geometry of the gasket in the flow chamber, 1 cm (width)×2.3 cm (length)×250 µm (height) [1], [9]. For each sample studied, the flow chamber was fixed in the same position during all μ-PIV tests.

**Figure 1:**
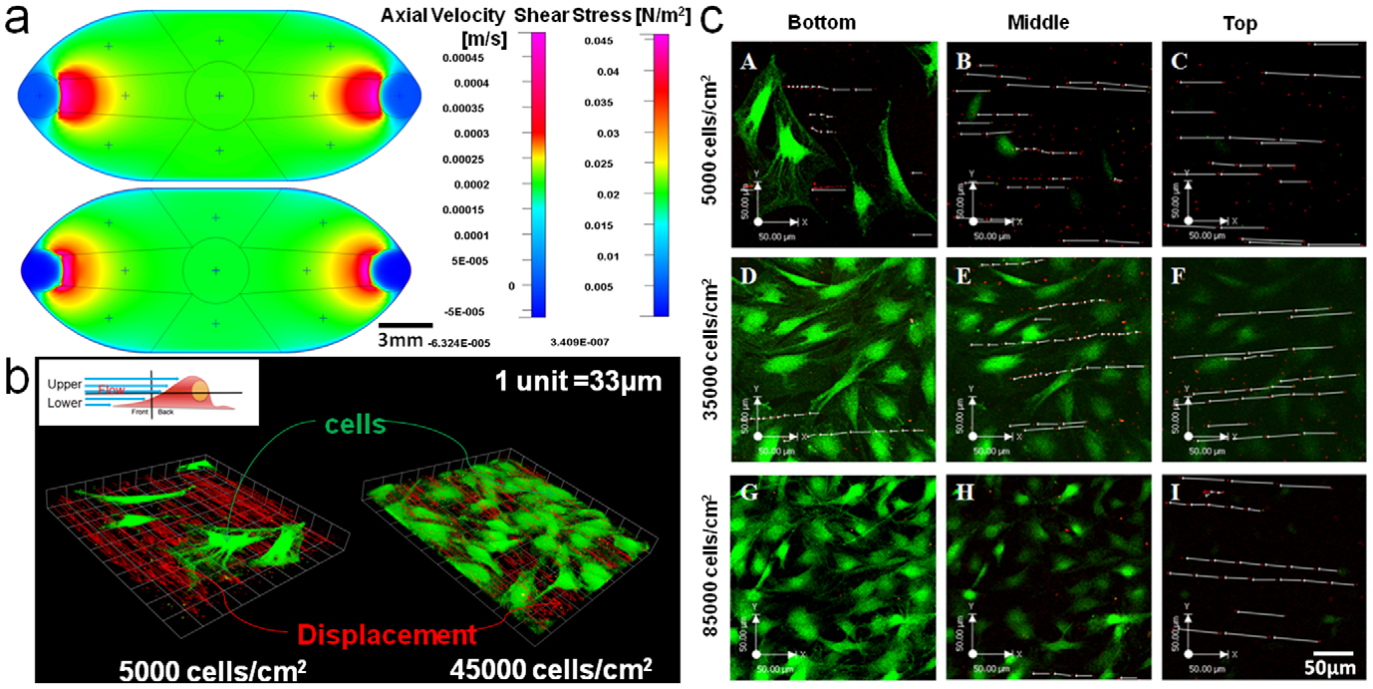
CFD predictions and μPIV for flow regimes of interest. (a) CFD predictions of axial flow velocity (upper) and shear stress (lower) in the flow chamber at 10 µm from the bottom of the flow chamber. Flow moves from right (inlet) to left (outlet). (b) 3D images of flow fields (red arrows indicate microsphere displacements) around cells (green). Three dimensional confocal image stacks were analyzed to quantify the flow fields with respect to distance from the substrate and cell density. (c) Confocal images close to the basal surface, approximately 2 µm from the substrate on which cells are seeded (A,D,G), 5 µm from the substrate (B,E,H), and 10 µm from the substrate (C,F,I). Cells are labeled with calcein green, microspheres exhibit red fluorescence, and white arrows indicated microsphere displacements in 990 milliseconds. Green, red, and white indicate cells, microspheres, and displacements, respectively.

Confocal images were taken within a 4 mm×4 mm area defined by microscope fields of view chosen as representative for each density group (40× objective, SP2 laser scanning confocal microscope, Leica Microsystems, Mannheim, Germany). Images were acquired, at heights from 0–10 µm from the substrate, at 256×256 resolution over 990 ms (Fig. 1c, Supplementary Video S1). Microsphere displacement was tracked using Image J (National Institutes of Health) and measured in space and time using image analysis software (Volocity, Improvision) (Fig. 1b, Supplementary Video S1).

### Statistical Analysis

First, mean particle displacements and velocities were measured for each image of a given image stack. Then, mean flow velocities were calculated for each layer, calculated from five samples at each cell density. Mean flow velocities and standard errors were plotted. A linear regression technique was applied to the data to allow for best comparison with computational predictions. JMP was used for ANOVA and Tukey's HSD (Honestly Significant Difference) tests for comparing velocities and shear stresses of each pair of densities and heights.

## Results

### In the absence of cells, CFD Predicts flow regimes within 12% of μ-PIV Measures

**Figure 2:**
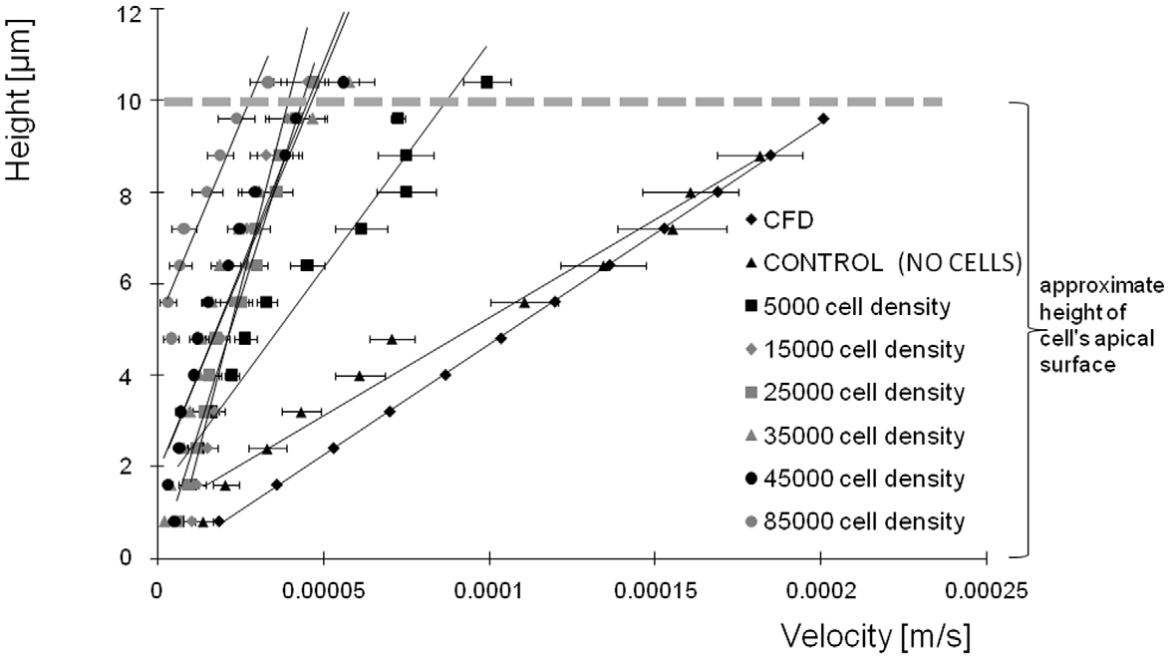
CFD predictions compared to experimental measures (μ-PIV) of velocity in the flow chamber, in the absence of cells. Error bars report standard error (n = 5) at each data point. All linear regressions show R^2^>0.8. The slopes of the linear regression lines represent the inverse of strain rate.

Computational fluid dynamics (CFD) predicts an optimized flow rate of 0.13 ml/min to achieve the 0.2 dyn/cm^2^ target shear stress in the circular target area at the center of a flow chamber, where the flow velocity and shear stress should be most uniformly distributed based on the design of the chamber [1] (Fig. 1a). Shear rates predicted by CFD and measured using μ-PIV are compared, since target shear stress is a function of shear rate for a Newtonian fluid [Equation 3]. For μ-PIV validation of CFD data, we compare shear rates at 10 µm from the bottom of the chamber (Fig. 1a), corresponding to the approximate mean cell height for our model embryonic mesenchymal stem cell line (Fig. 1b,c). Furthermore, since slopes of data points in Fig. 2 represent the inverse of shear rate, we calculate the mean shear rate and standard error from linear regressions of the control data. Based on μ-PIV data, the mean shear rate is 21.02±2.23 (s.e.m.) [sec^−1^]. Comparing experimental data with CFD predictions, there is a 0–11.13% difference in the shear rate predicted by CFD and the shear rate measured by μ-PIV. Hence, without the inclusion of cells within the flow channel, CFD predicts flow regimes to within 12% of experimentally measured values.

### The presence and density of cells significantly affects flow

**Figure 3:**
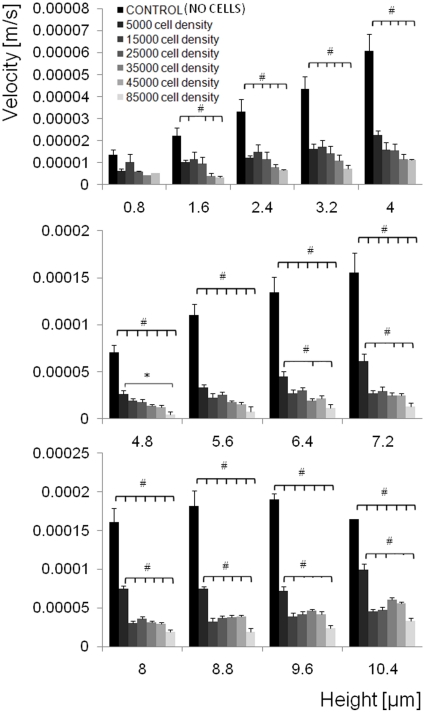
Velocity (m/s) as a function of height (µm) from bottom of flow chamber. *, # Statistical significance is defined by p<0.05. Error bars depict standard error of the mean (s.e.m.). From 4.8 µm to 7.2 µm at VHD, microparticels were observed at only two or three experiments out of five experiments.

The presence of cells along the base of the flow chamber significantly influences flow induced shear stresses that define the mechanical milieu of the cell. Furthermore, the changes in flow patterns attributable to the presence of cells relate significantly to the density of cell seeding and the height from the basal surface at which flow is assessed. Cells from the model embryonic mesenchymal stem cell line C3H/10T1/2, seeded at 5,000 cells/cm^2^ (very low density, VLD), 15,000 and 25,000 cells/cm^2^ (as low density, LD), 35,000 and 45,000 cells/cm^2^ (high density, HD), and 85,000 cells/cm^2^ (very high density, VHD) significantly influence the flow field compared to the flow field in absence of cells (p<0.05). In general, with increasing cell seeding density, the velocity of flow as well as flow induced shear rate (strain rate) decrease (Fig. 2). Furthermore, the influence of cell seeding density on velocity varies significantly with height from the bottom of flow chamber (Fig. 3), where differences between seeding density groups are greatest in the nonvicinity region (above 4 µm from the cell's basal surface). Among low density seeding groups (VLD, LD) significant differences in flow velocity are also apparent closer to the apical surface of the cell (4.8–10.4µm above the basal surface). Finally, at VHD, few microspheres are observable in the vicinity of the cell's basal surface, since cells are packed so closely that fluid cannot flow between cells.

### Shear stress varies significantly along the vertical profile of the cell

**Figure 4:**
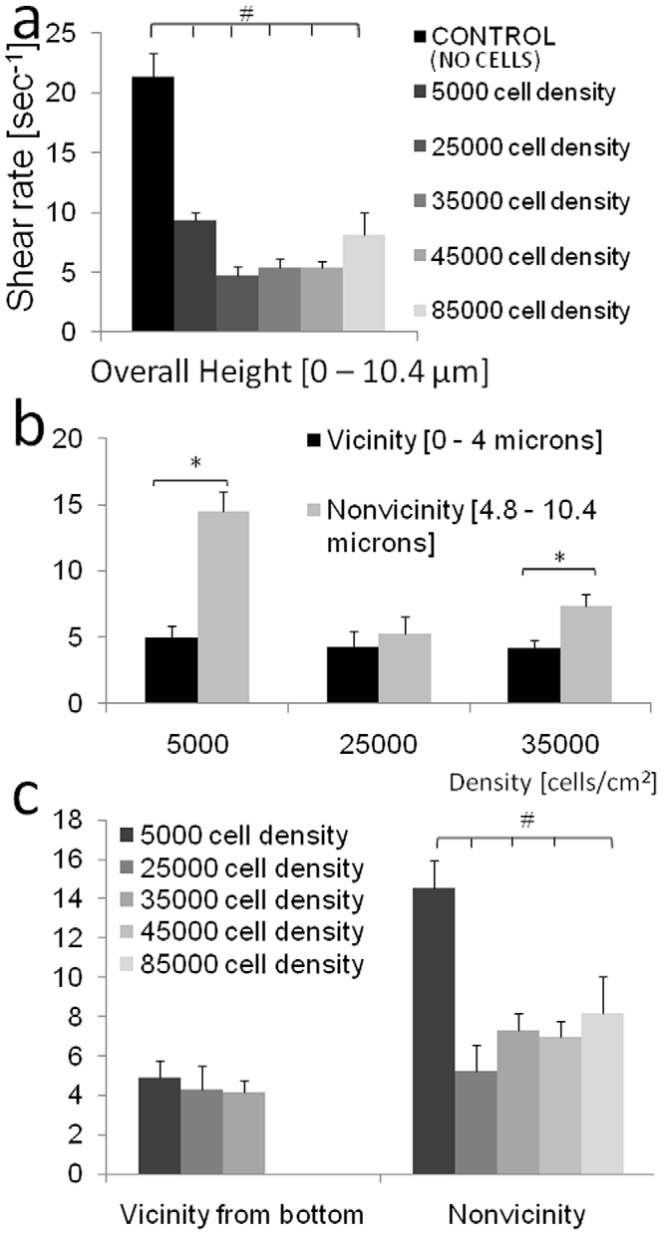
Shear stress variation as a function of cell height and cell seeding density. (a) Shear rate (linear slope at each density, from Fig. 2) as a function of height, from the base of the chamber to 10.4 micron from the base of the chamber. Statistical significance is shown for p<0.05. (b,c) Shear stress variation with respect to cell density and height from the base of the chamber. Shear rate increases along height for 5,000, 25,000, and 35,000cells/cm^2^ (b). Density effects on shear rate with respect to height from the base (c).

Shear stress varies significantly along the vertical profile of the cell due to the variance in velocity of flow with respect to height from the cell's basal surface (Fig. 4a). In areas toward the apical surface of the cell (4.8–10.4 µm above the basal surface) significant differences in shear stresses are observable between the two low density groups (VLD, LD) and the VLD and HD groups. In areas near (0–4 µm from) the basal surface of the cells and between all other groups, no significant differences in shear stress are observed (Fig. 4b,c).

## Discussion

These studies demonstrate, for the first time to our knowledge, the use of μ-PIV measures to map the live stem cell's mechanical milieu, spatially, temporally and at the subcellular scale. In the absence of cells, CFD predicts flow regimes within 12% of μ-PIV measures. Hence, CFD predicts flow regimes accurately for the technical specification of the chambers and the flow rates necessary to deliver target stresses at a particular height from the base of the chamber. However, based on our μ-PIV studies, flow is significantly influenced by the presence of cells *per se* as well as by the density at which cells are seeded. Furthermore, for any given cell or cell seeding density, flow regimes vary significantly along the vertical profile of the cell. Hence, the mechanical milieu of the stem cell exposed to fluid drag induced, shape changing shear stresses, varies with respect to proximity of surrounding cells as well as with respect to apical height.

Interestingly, as observed in the companion study, cell seeding density exerts a profound influence on the local mechanical milieu of cells exposed to controlled fluid drag-induced shear stresses, which appears to relate directly to the mechanoadaptation response observed after exposure to flow. While low (5,000 cells/cm^2^) and high (35,000 cells/cm^2^) density seeding protocols are designed to achieve nonconfluent and confluent states, respectively, to emulate different development stages in the context of the first stage of skeletogenesis, three dimensional flow field measurements carried out in the current study demonstrate the profound influence of neighboring cells on flow regimes and hence local shear stresses to which cells are subjected. Based on the results of the companion study, after 30 minutes of flow, cells seeded at high density appear to have adapted anisotropically to distribute stresses evenly at cell-fluid interfaces. Interestingly, we observed that cells seeded at low density have a significantly higher stress toward the apical half of the cell than cells seeded at the higher density, which may reflect a less robust anisotropic adaptation of these cells as observed in the companion study. Hence, taking into context our companion study showing the mechanical adaptation of stem cells to their dynamic local milieu [10], it appears that stem cells may ultimately exhibit the capacity to modulate their own environment.

Given the complexity of cell-cell interactions and the novelty of mapping 3D flow fields in live cells at different seeding densities, we should take several limitations of the current study into account when designing future studies. The current study does not account for potential effects of cell-cell junctions, which may redistribute force balances at boundaries of densely seeded cells. In addition, it does not account for potentially mitigating slipstream effects, where downstream cells are buffered from flow [11], [12]. Accounting for these biological and flow effects in future studies may provide further insight into the interplay between mechanoadaptation of cells and mechanomodulation by cells.

These studies have important implications not only for *de novo* engineering of functional tissues from stem cell templates but also for understanding the underlying mechanisms of multiscale functional adaptation. Whereas biochemical cues that steer stem cells toward osteogenic, myogenic, and chondrogenic [13]–[20] lineages have been described in detail, more recent studies point toward a crucial role for biophysical signals in cell lineage determination [21]–[25]. Pauwels postulated that dilatational, volume changing stresses (tension, compression) steer stem cells toward chondrogenic lineages and that deviatoric, shape changing stresses (shear) steer stem cells toward ligamentous and tendonous lineages [25]. However, neither the mechanical signals experienced by multipotent progenitor cells during development nor the specific nature of mechanical signals conducive to guiding pluripotent cells toward specific lineages are well characterized [2]. *In vitro* studies have shown that such mechanical loading significantly affects terminally differentiated cells as well as lineage commitment in undifferentiated multipotent stem cells [3], [26]–[44]. Furthermore, mechanical stimuli influence tissue morphogenesis, and embryonic mesenchymal stem cells exhibit approximately a thousand times greater mechanosensitivity than terminally differentiated cells [1], [2]. Computational models provide a platform to predict and simulate mechanical forces in cell morphogenesis [21], [23], [24] and mechanotransduction [45], [46]. Comparative studies using CFD have underscored the need for an understanding of the cell's mechanical milieu in three dimensions [6]. The current study addresses that previously unmet need, in which we predict flow regimes and observe mechanoadaptation of cells in flow chambers designed to deliver precisely controlled mechanical signals to live cells [1]. Furthermore, microscale particle image velocimetry (μ-PIV) allows not only for validation of CFD predictions at the length scale of the coverslip onto which cells are seeded but also for observation of cell seeding effects on the three dimensional flow field in the vicinity of cells.

The role of seeding density and/or number and proximity of neighbors on the local mechanical milieu of the stem cell is of particular interest not only for *de novo* tissue engineering during development or healing of tissue defects [47], but also in the context of seeding tissue engineering scaffolds with stem cells. CFD has been used to understand and optimize the use of tissue engineering scaffolds as delivery devices for biochemical and mechanical cues [2]. Not only do three dimensional CFD studies within scaffolds allow for accurate prediction to flow regimes inside of the scaffold, but also 3D CFD can be used in concert with the μ-PIV approach described here to optimize cell seeding protocols and delivery of appropriate mechanical cues for targeted tissue design and manufacture, as well as to understand and control degradation of biodegradable tissue scaffolds. Finally, using CFD models, we can predict and optimize the *in vivo* mechanical milieu of cells seeded within scaffolds after implantation in the body.

The role of the cytoskeleton is to balance and to transduce intrinsic and extrinsic mechanical forces [48]–[50]. Comprised of elements, *e.g.* actin filaments, microtubules, intermediate filaments, which themselves show subspecialized mechanical functions [50], we expect to see different mechanoadaptive responses in response to shape changing deviatoric or volume changing dilatational stresses [9]. Given the results of our companion studies showing the mechanical adaptation of stem cells to their dynamic local milieu and these current studies demonstrating the effect of cells themselves on local flow fields, it appears that stem cells may ultimately exhibit the capacity to modulate their own environment by altering their structure, which redistributes forces at cell-environment interfaces. Furthermore, at a higher length scale, cells within multicellular structures modulate force balances at boundaries not only through adaptation of their own architecture but also through specialization of higher order structure to function in the prevailing mechanical environment. Hence, an understanding of interactions and adaptation in response to forces at the interface between the surface of the cell and its immediate local environment, *i.e.* the elucidation of emergent cell anisotropic architecture, may be key to unraveling the mechanisms underlying multiscale development, growth and adaptation of organisms.

## Supporting Information

Video S1 Three dimensional flow (3D flow) is highly dependent on the proximity of cells and distance from the base of the chamber (basal cell surface). 3D flow is visualized using red fluorescent microspheres, taken as a stack of planar confocal images, from the base of the flow chamber up to 10 microns height. Red arrows depict flow vectors in space, i.e. the displacement (arrow length) and direction (arrow head direction) of flow over approximately 1 second are depicted for single microspheres of 1 micron diameter. Cells, seeded at a density of 15,000 cells/cm^2^, are labeled with calcein green stain.
(0.21 MB AVI)
